# The COVenT study: developing a core outcome set for mechanical ventilation trials

**DOI:** 10.1186/1745-6215-16-S1-P35

**Published:** 2015-05-29

**Authors:** Bronagh Blackwood, Suzanne Ringrow, Danny McAuley, Mike Clarke

**Affiliations:** 1 School of Medicine, Dentistry and Biomedical Sciences, Queen’s University Belfast, BT7 1NN Northern Ireland, UK

## Background

Inconsistency in trial outcomes reporting is a widely recognised problem in clinical research and critical care research is no exception. Our 2014 review of 66 trials evaluating reporting of ventilation outcomes, revealed a significant heterogeneity in both the outcomes selected and their definitions, particularly in terms of start and end points [1].

The purpose of our study is to establish a core outcome set (COS) for trials of interventions which aim to modify the duration of mechanical ventilation. We will actively promote the use of this set and follow its uptake in the academic press for ten years post publication.

## Methods

The study will begin with an online, international Delphi and close with a smaller face-to-face consensus meeting. We have identified seven key stakeholder groups to participate; intensive-care physicians, nurses, trial groups, investigators, industry, funders and ambassadors for patient organisations.

The Delphi has 3 sequential rounds of questionnaires asking participants to score each outcome depending on perceived importance for inclusion in the COS. Collated scores from previous rounds are forwarded anonymously to participants so to prompt reflection and establish consensus. The initial set of outcomes will be drawn from our systematic review and participants may add any others they feel are relevant. Using a Delphi provides an opportunity to investigate how the manner of feedback that participants receive affects the end consensus. To do this we will randomise the panel (stratified by stakeholder group) into 3 groups each receiving feedback in different forms and assess the resulting outcomes sets for homogeneity.

The final COS will be determined at the consensus meeting following presentation and discussion of the Delphi.

## Conclusion

The COVenT study will present the first COS for ventilation and will provide new insight into the methodology of the Delphi technique. We hope to complete this project by December 2015.
